# Phenology drives species interactions and modularity in a plant - flower visitor network

**DOI:** 10.1038/s41598-018-27725-2

**Published:** 2018-06-20

**Authors:** Javier Morente-López, Carlos Lara-Romero, Concepcion Ornosa, José M. Iriondo

**Affiliations:** 1 Biodiversity and Conservation Area, School of Experimental Sciences (ESCET), Rey Juan Carlos University, Madrid, Spain; 2 Global Change Research Group, Mediterranean Institute of Advanced Studies (CSIC–IUB), Esporles, Mallorca, Spain; 3 Departamento de Biodiversidad, Ecología y Evolución, Complutense University, Madrid, Spain

## Abstract

Phenology is often identified as one of the main structural driving forces of plant – flower visitor networks. Nevertheless, we do not yet have a full understanding of the effects of phenology in basic network build up mechanisms such as ecological modularity. In this study, we aimed to identify the effect of within-season temporal variation of plant and flower visitor activity on the network structural conformation. Thus, we analysed the temporal dynamics of a plant – flower visitor network in two Mediterranean alpine communities during one complete flowering season. In our approach, we built quantitative interaction networks and studied the dynamics through temporal beta diversity of species, interaction changes and modularity analysis. Within-season dissimilarity in the identity of interactions was mainly caused by species replacement through time (species turnover). Temporal replacement of species and interactions clearly impacted modularity, to the extent that species phenology emerged as a strong determinant of modularity in our networks. From an applied perspective, our results highlight the importance of considering the temporal variation of species interactions throughout the flowering season and the requirement of making comprehensive temporal sampling when aiming to build functionally consistent interaction networks.

## Introduction

Biotic relationships create complex networks involving great number of species that represent the assembly of the community^1,2^. As community dynamics greatly depend on the way species interact, the resulting interaction networks conform the “biodiversity architecture”^1^. Biotic interaction networks play an important role in the stability of ecosystems^3^, as well as in the maintenance of global and local biodiversity^4^. Complex network analysis has become an important tool for interaction studies because it provides information on community organization and help to predict community dynamics in response to ecosystem disturbance^3,5,6^.

Plant – flower visitor networks are normally analysed and studied using all the interactions recorded in the community throughout the whole flowering season. This approach allows the analysis of the network as a whole, characterizing the system in a general way with a single set of parameters and provides information on the pairs of species interacting in the network. Although the network analysis integrating the whole flowering season is a key approach to understanding community-wide patterns of plant – flower visitor interactions, the information that it provides is limited by the integration and may be misleading if not interpreted correctly^7^. One simple limitation is that it cannot assess the structural dynamism of the network throughout the flowering season^8,9^. Furthermore, it does not assess if the recorded interactions concur in time and take place simultaneously, take place with a partial overlap or at completely different time intervals. Consequently, it is not possible to infer if the different pollinators that visit a particular plant species are competing at the same time for the same resources or visit the plant in a sequential order, and vice versa^7^. This synthetic approach cannot interpret the lack of interactions between pairs of species, whether they do not take place due to lack of temporal synchrony between flowering time and the adult stage of the insect, to incompatibilities between flower and insect morphologies, or to insect preferences^10^.

Phenology and morphological variability of the different species involved in networks during the flowering season have been identified as the main structural driving forces of plant – flower visitor networks^10,11^. Nevertheless, we do not yet know the specific effects of phenology in basic network build up mechanisms such as ecological modularity. Modularity describes the relative strengths of sets of interacting species and provides insights into the dynamics of ecological interactions. In modular networks subsets of species interact more frequently with each other than with species in other modules^12,13^. Although there is ample empirical evidence indicating that modularity may be driven by spatial or habitat segregation, trophic specialization, divergent selection regimes, and phylogenetic clustering of closely related species^13–15^, the role of species phenology in the structure of this key property has been rarely explored^9,14^. As modularity is expected to increase with link specificity of all species, it may also be driven by flower-visitors and flowering phenology, which may generate non-overlapping phenophases between interacting mutualists, and hence determine the availability of species interactions in the network^10^.

The low temperatures that reign in alpine communities for most of the year constrain physiological activity to a very short period between the start of snow melt in spring and the arrival of snow in autumn^16^. The length of this period in Mediterranean alpine communities is further limited by the lack of rainfall in summer, which originates a mid-summer drought that seriously hampers growth and reproduction^17,18^. Consequently, the length of the flowering season in these plant communities is very short and variable^6,19^. Furthermore, insect and flowering phenology is under selection and affected by changes in climate conditions^17,20,21^ such as those produced by global warming. In this scenario, it is essential to understand the role of insect and flowering phenology on plant-pollinator interaction dynamics.

**Figure 1:**
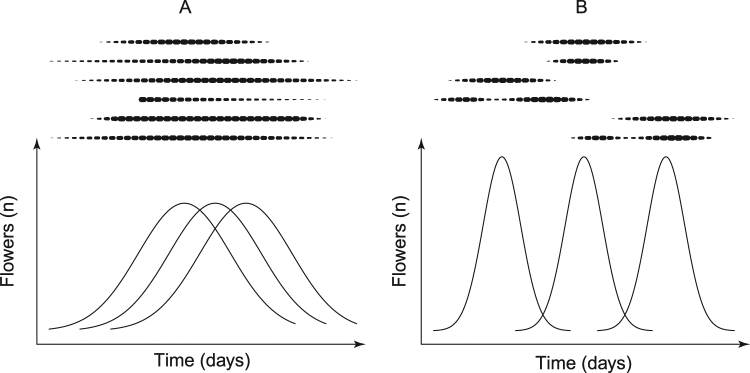
Conceptual figure representing two contrasting scenarios of phenological coupling among plants (solid lines) and flower-visitors (dashed lines) across a flowering season. Panel (A): the harsh abiotic conditions of alpine environment (e.g., late spring frosts, early autumn snowfall) force the staggering of flowering times to a minimum. As a result, the flowering peak of most plant species would coincide within a short period of time and most interactions of the network would be concentrated in this period. Panel (B): the flowering phenologies spread along the short season to minimize competition for pollinators. As a result, insect and flowering phenologies would be temporally segregated into different groups, which would result in a temporal replacement of species and interactions. Each solid and dashed line represents a hypothetical species.

In this study, we aimed to explore the within-season temporal dynamics of plant-flower visitor interactions and to assess the effect of phenology on the network structural conformation. We analysed the temporal dynamics of a plant - flower visitor network in a Mediterranean alpine community during one complete flowering season. In our approach, we built quantitative interaction networks along the season and studied the dynamics through beta diversity of species, interaction changes and modularity analysis. The short period of activity in Mediterranean alpine communities provides a simple reference baseline to study the within-season temporal dynamics of plant – flowering visitor networks. In these circumstances, we hypothesized that the flowering period would be short and the flowering peak of most plant species would coincide within a short period of time, instead of being staggered to reduce competition^22^. Consequently, most network interactions would be concentrated in this period (Fig. 1A), instead of temporally segregated into different groups (Fig. 1B). Furthermore, we expected temporal replacement of species to be constrained by the short flowering period of the community, thereby preventing the formation of modules of plants and flower visitors associated with temporal variation in the interactions.

## Results

### Within-season variation in the composition of species assemblages and interactions

**Figure 2:**
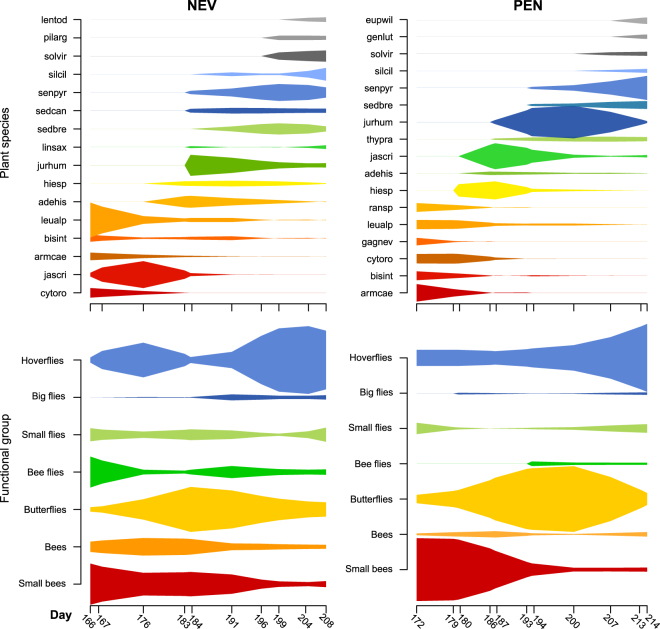
Within-season variation in the activity of plant species and the main functional groups of flower visitors at Nevero (NEV) and Peñalara (PEN) study sites. The width of the spindle diagrams denotes the number of visits of each plant species and each functional group. Note y-axes and colours used for plant species are not the same in NEV and in PEN.

A total of 103 flower-visitors and 17 plant species at PEN and 115 flower-visitors and 16 plant species at NEV were recorded (Table S1). The total number of visits recorded at PEN was higher than at NEV (3278 and 2261, respectively), but the number of interactions was lower at PEN than at NEV (240 and 315, respectively). The flowering periods of the study species were diverse, with few complete overlaps (Fig. 2 & Fig. S1; interspecific flowering overlap, mean ± SD: 0.42 ± 0.32 at PEN; 0.44 ± 0.29 at NEV), and short blooming periods (mean ± SD: 20 days ± 8 at PEN; 20 days ± 7 at NEV). Overall, the forb species *Jasione crispa*, *Jurinea humilis* and *Senecio pyrenaicus* together with the shrub species *Cytisus oromediterraneus* and *Adenocarpus hispanicus* accounted for 52% of all links and 69% of all visits at PEN and 49% and 58% at NEV. In any case, most plant species were highly connected. 65% of the plants at PEN and 81% at NEV had at least 10 flowering visitors with 31 or more visits (for further details on each plant species, see Table S1).

The main functional group of flower visitors, according to their visitation activity, changed throughout the flowering season at both study sites (Fig. 2). Overall, large and small solitary bees (order: Hymenoptera) were the predominant flower visitors at the early stage of the flowering season, while butterflies (Lepidoptera) and hoverflies (Diptera) were more active at the mid and late stages (Fig. 2). These four functional groups of flower visitors accounted for most interactions and visits in both networks. Thus, they accounted for 57% of interactions and 71% of visits at PEN and up to 54% of interactions and 68% of visits at NEV.

**Figure 3:**
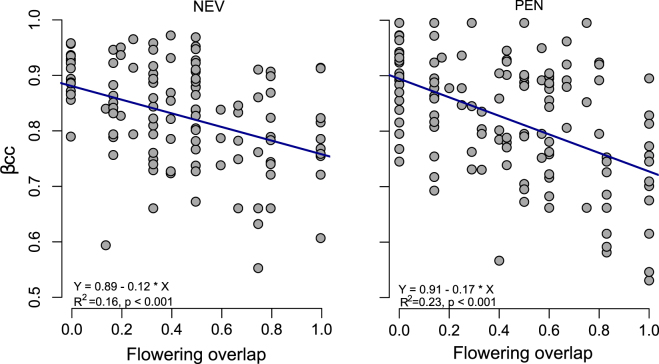
Relationship between inter-specific flowering overlap and Jaccard dissimilarity (β_cc_) of flower-visitor assemblages.

**Figure 4:**
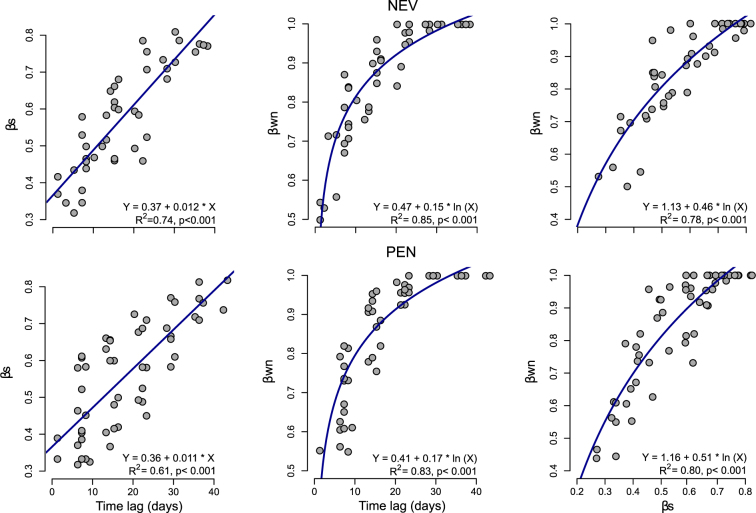
Relationship between dissimilarity in species composition (β_S_) and interactions (β_WN_) and time lag between daily sub-networks. β_S_ and β_WN_ were estimated using Whittaker (1960) as measure of dissimilarity. For β_WN_, data are plotted on the original scale, but statistics are from model fit to log-transformed data.

Overall Beta diversities, measured as Jaccard dissimilarity (β_CC_) of plant and flower-visitor assemblages were similar at both study sites and ranged between 0.85 and 0.89 (Table 1). The decomposition of dissimilarity patterns in species assemblages into replacement (β_3M_) and richness (β_RICH_) components showed that a high proportion of species experienced temporal replacement since β_3M_ represented between 73 and 85% of overall beta diversity of plant and flower-visitor assemblages (Table 1). A negative linear relationship between inter-specific flowering overlap and β_cc_ of flower-visitor assemblages at both study sites was found (Fig. 3)_,_ which implies that flower-visitors’ assemblages were more similar between species with more synchronous flowering and vice versa. Temporal replacement of species was also evidenced by the significant positive linear relationship between temporal distance among daily sub-networks and dissimilarity in species composition (β_S_) (Fig. 4). As a result, interaction turnover (β_WN_) also showed a clear temporal pattern as it increased with time lag among sub-networks (Fig. 4). Indeed, there was a complete turnover of interactions after ~20 days in the two sites, *i.e*., when β_WN_ reaches its maximum (asymptote) value due to complete change of plant species assemblages. The contrast between β_S_ and β_WN_ also followed a positive log-linear relationship as β_WN_ rapidly increased until β_S_ reached intermediate values (i.e., 0.4–0.6), a point at which β_WN_ reduces its growth until the maximum value is reached (Fig. 4).

**Table 1:** Within-season beta-diversity of plant and flower visitor assemblages at Nevero (NEV) and Peñalara (PEN) study sites.

	β_CC_	β_3M_	β_RICH_
**Plant species assemblages**			
NEV	0.85	0.62	0.23
PEN	0.87	0.72	0.15
**Flower visitor assemblages**			
NEV	0.89	0.76	0.13
PEN	0.89	0.65	0.24

Beta-diversity was analysed among the daily networks of interaction recorded for each study site.

Β_CC_ is overall Jaccard dissimilarity. β_3M_ and β_RICH_ are replacement and richness components of Jaccard dissimilarity, respectively.

### Temporal component of modularity

The cumulative plant–flower visitor networks of both study sites displayed a significant modular structure (NEV, *Q* = 0.34; PEN: *Q* = 0.26; Z-test: both *p* < 0.001). Five and three modules were identified at NEV and PEN, respectively (Table 2, Fig. S3). The species composition of each module is listed in the electronic Supplementary Material (Table S1).

**Table 2:** Distribution of plants and number of flower-visitors of the modules identified for Nevero (NEV) and Peñalara (PEN) study sites.

	NEV					PEN		
	Modules					Modules		
	NEV 1	NEV 2	NEV 3	NEV 4	NEV 5	PEN 1	PEN 2	PEN 3
**Plant species assemblages**								
	armcae	bisint	sedbre	linsax	solvir	ransp	hiesp	silcil
	adehis	jascri	sedcan	lentod	—	bisint	genlut	solvir
	leualp	silcil	—	pinvah	—	thypra	jascri	adehis
	jurhum	hiesp	—	—	—	armcae	cytoro	sedbre
	cytoro	—	—	—	—	gagnev	jurhum	senpyr
	senpyr	—	—	—	—	—	leualp	eupwil
**Flower-visitor assemblages**								
Bee Flies	1 (16.7)	—	4 (66.7)	1 (16.7)	—	1 (20)	1 (20)	3 (60)
Bees	10 (66.7)	5 (33.3)	—	—	—	4 (30.8)	6 (46.2)	3 (23.1)
Beetles	9 (81.8)	—	—	—	2 (18.2)	3 (27.3)	5 (45.5)	3 (27.3)
Bumblebees	1 (33.3)	2 (66.7)	—	—	—	—	2 (100)	—
Butterflies	17 (73.9)	5 (21.7)	1 (4.3)	—	—	6 (23.1)	16 (61.5)	4 (15.4)
Flies	5 (71.4)	—	—	—	2 (28.6)	1 (12.5)	4 (50)	3 (37.5)
Hoverflies	5 (38.5)	1 (7.7)	4 (30.8)	—	3 (23.1)	1 (7.7)	3 (23.1)	9 (69.2)
Others	8 (80)	2 (20)	—	—	—	2 (22.2)	2 (22.2)	5 (55.6)
Small Bees	2 (20)	3 (30)	—	3 (30)	2 (20)	2 (33.3)	4 (66.7)	—
Small flies	9 (90)	1 (10)	—	—	—	1 (20)	3 (60)	1 (20)
Wasps	4 (57.1)	—	2 (28.6)	—	1 (14.3)	1 (20)	1 (20)	3 (60)

Percentage of species with respect to the total of each functional group is given in parentheses. Modules are named according to their peaks of activity. Species identities in each module and acronyms used for plant species are given in the electronic Supplementary Material, Table S1.

**Figure 5:**
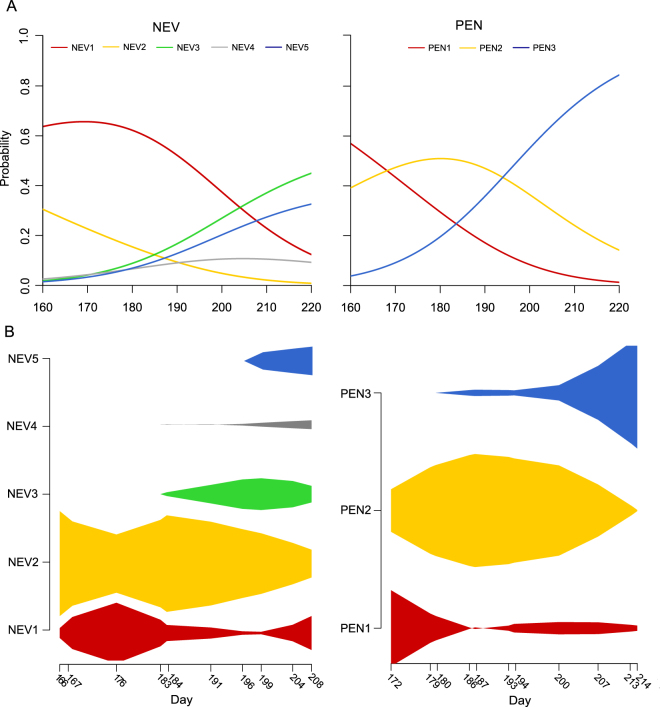
Distribution of activity within identified modules at Nevero (NEV1- NEV5) and Peñalara (PEN1-PEN3) study sites. (**A**) Predicted probabilities of module membership based on species phenology obtained from multinomial logistic regression analysis. (**B**) Within-season variation in activity of the modules. The width of the spindle diagrams denotes the relative activity (i.e., visits recorded for plant species) of each module.

The activity of the modules (measured as total visits received by the plants of each module) was not homogeneously distributed over time at either study site (Fig. 5B; one-way χ^2^-tests: all modules *p* < 0.001). At PEN, module PEN2 was active throughout the entire sampling period but concentrated the bulk of its activity at the mid stage of the flowering season (Fig. 5B). On the other hand, modules PEN1 and PEN3 were mainly active at the beginning and end of the flowering season, respectively (Fig. 5B). At NEV three modules were only active in the second half of the flowering season (Fig. 5B: see modules NEV3, NEV4 and NEV5), while the activity of modules NEV1 and NEV2 was concentrated in the first half but also extended into the second half (Fig. 5B). Multinomial logistic regressions showed that start date of species activity significantly explained how species were arranged in different modules (Fig. 5A, Likelihood Ratio *χ*^2^ tests: NEV: *χ*^2^ = 23.4, *df* = 4, *p* = 0.0001; PEN: *χ*^2^ = 26.42, *df* = 4, *p* < 0.0001). In NEV, species with early phenologies had higher probability to be assigned to two modules (NEV1 and NEV2), while other modules (NEV3 to NEV5) tend to harbour species that start their activity at the mid- and end-stage of the flowering season (Table S2, Fig. 5A). In PEN, species that start their activity at the early- and mid-stage of the flowering season were more likely to former modules PEN1 and PEN2 respectively, while PEN 3 tend to be formed by species with late phenologies (Table S2, Fig. 5A).

The distribution of functional groups of flower-visitors had an evident temporal and phylogenetic component (Table 2). Hence, functional groups from the Order Hymenoptera (*i.e*., small bees, bees and bumblebees) mainly formed modules that had their peak of activity at early and mid-stages of the flowering season (*i.e*., PEN1, PEN2, NEV1, NEV2). Most butterflies (Lepidoptera) and beetles (Coleoptera) took part in modules with peak activity in the early- mid-flowering season (PEN2 and NEV1), while hoverflies (Diptera) tended to conform modules that were active late in the season (PEN3, NEV3, NEV4).

## Discussion

Despite the short period of activity in Mediterranean alpine communities, within-season variation in the composition of plant and flower-visitor assemblages was wide (β_CC_). It was mainly driven by the temporal species turnover component (β_3M_) related to species replacement through time rather than by the richness component (β_RICH_). Within-season variation of plant-flower visitor interactions was also high, and increased with temporal distance among daily sub-networks. Temporal replacement of species and interactions clearly impacted modularity.

### Within-season variation in the composition of species assemblages and interactions

Almost all plant species displayed many flowering individuals over a short period of time in the flowering season (Fig. 1B), rather than flowering for a longer period and overlapping with other flowering species as predicted (Fig. 1A). Flower visitors also showed a clear temporal pattern in the relative occurrence of their visits. Large and small solitary bees predominated at the early stage of the flowering season, while the proportion of butterflies and hoverflies increased throughout the flowering season. The main mechanism responsible for this fluctuation was species replacement as shown by the decomposition of beta diversity measures. This indicates that temporal replacement of species was not constrained by the short period of activity at the study sites, as we initially hypothesized (Fig. 1A). This also explains the negative relationship found between inter-specific flowering overlap and dissimilarity in flower-visitor assemblages. As a result, the long-term persistence of interactions was rare, as evidenced by the strong positive relationship between dissimilarity in species composition (β_S_), interactions (β_WN_) and time lag between daily sub-networks. Hence, interaction turnover was primarily driven by phenological changes (species functional availability) throughout the growing season. Instead the contribution of re-wiring appears to be low as suggested by the strong relationship between β_S_ and β_WN_. Yet, there were not plants species shared between subnetworks separated more than 28 days, revealing a phenological disconnection.

We believe that our sampling effort was adequate and that the methodological approach is robust enough to provide an accurate picture of the internal temporal processes of the network. In any case, it should be noted that if some species were present but undetected on one or more census days, the sampling bias might somewhat inflate species replacement rates^23^. To evaluate that, we conducted beta-diversity analysis with each aggregation time-window spanning two consecutive census days and a calendar week. β_3M_ fell slightly compared to previous analyses but remained well above β_RICH_ (Table S3). β_WN_ and β_S_ also maintained their positive relationship with temporal distance among sub-networks (Fig. S2) and the phenological disconnection caused by the effect of temporal species replacement remained. Hence, these additional analyses confirmed within-season temporal dynamics of plant – flowering visitor interactions and supported the robustness of our approach. Furthermore, results are consistent with those of previous studies that have documented strong variation in the composition of species and interactions within the same season for communities with extended^14,24–26^ and short activity periods^8,27,28^. In our case, we found that within-season variation of interactions was dominated by species replacement via phenological processes. This is a noteworthy result because contribution of re-wiring towards interaction was described as an inter-annual property of flower-visitor foraging that does not frequently occur at the time scales herein^28–30^.

### Temporal component of modularity

Contrary to our expectations, the short vegetative period did not prevent the formation of modules associated with temporal variation in the interactions. Instead, temporal replacement of species and interactions restricted species connectivity patterns^10,11,27^. This contributed to the observed modularity that was structured as a sequence of modules acting at different times across the flowering season. Consequently, species phenology showed a strong association with the detected modular structure, as inferred from the significance of the start date of species activity on module membership of the species. Furthermore, we described a phenological pattern consistent with the visitation activity of flower visitors, with early-stage modules formed mostly by hymenopterans, an important irruption of butterflies in the mid-stage modules, and a preponderance of hoverflies in the late-stage modules. Taken together, these findings strongly support that species phenology is a strong determinant of modularity in these networks. There is considerable literature showing that species abundance and phenological overlaps seem to play a key role in structuring mutualistic interactions (e.g.,^10,11,30,31^). Particularly for modularity, the few studies that have examined the role of species phenology in structuring the modularity of pollination networks^9,14,32^ provided similar results to those reported herein. Furthermore, the formation of temporal modules comprising birds and plant species with phenological overlap have also been reported in plant-frugivore interaction networks^33,34^.

Regarding species composition, modules have been viewed as potential coevolutionary units of biological significance^13–15^. The formation of modules comprising plant species flowering at about the same time may be the result of selection against overlapping flowering phenophases, which could help to ensure pollination by minimizing inter-specific competition for shared pollinators^6,11,28^. From the perspective of flower visitors, phenological uncoupling between functional groups would suggest temporal resource partitioning based on behavioural or morphological variation^35^. For many species, phenological uncoupling probably represent differences in migratory timing or local population cycles^24,36^. It is important to note that modularity was not completely explained by displacement of plant phenologies as activity of temporally displaced modules partially overlap (e.g., NEV 1 and NEV2 and PEN 1 and PEN2). This implies the existence of other factors contributing to the observed modular conformation. These factors are probably related to preference of flower-visitors for certain plant species. For example, modules can also arise if a subset of plant species with overlapping flowering phenologies presents morphological and physiological characteristics that can attract or constrict certain groups of flower visitors over the others^10,25^. However, species flowering at about the same time displayed a diverse array of morphological and physiological traits and tended to be visited by similar flower-visitors, irrespective of their floral features (Table S1). Hence, results did not provide clear evidence in this regard, and therefore, the formation of temporal modules comprising functional groups of flower visitors requires further investigation.

Modular patterns are expected to increase overall network robustness, retaining the impacts of a perturbation within a single module and minimizing impacts on other modules^13^. At the same time, a strong dependence of species on a narrow set of interaction partners may render species more vulnerable to co-extinction^1,2^. In this context, these networks are vulnerable to the predicted phenological mismatches between plants and flower visitors that can arise with environmental change^37,38^. This may result in scarcity of floral reward supplies and pollinators throughout the period in which they are phenologically uncoupled, thus having a negative impact on population dynamics^37,38^. On the other hand, opportunities to build up new interactions would emerge as many flower-visitors will ‘rewire’ their ecological links in absence of missing partners, which may mitigate the consequences of shifts in phenology for many species^38^. In the face of increasing global warming in alpine environments world-wide^38^, we urgently need further empirical studies to measure the effects of climate change on the structure and temporal dynamics of their plant–flower visitor interaction networks.

## Conclusions

The short period of activity in Mediterranean alpine communities provided a simple reference baseline to study the within-season temporal dynamics of plant – flowering visitor interactions. Our findings revealed that within-season dissimilarity in the identity of interactions was mainly caused by temporal species replacement. More importantly, they also showed that plant and flower-visitors phenology can be a strong determinant of modularity in ecological networks. Given the results obtained in a community with a short period of activity, we expect communities with longer activity periods to have greater opportunities to establish interactions that are temporally segregated into different groups. Consequently, their temporal dynamics may be poorly understood when they are studied integrating the data of the whole flowering season. It should also be noted that a bias can be generated in a plant - flower visitor network construction if only a short period of the flowering season of the communities is considered in data collection. In essence, our results highlight the importance of analysing the within-season temporal variation of interactions in order to gain insight into the underlying mechanisms that determine network structure. The temporal component of modularity may entail functional consequences for the persistence and evolution of alpine communities, which must be thoroughly investigated in the face of climate change.

## Methods

### Study area

This study was carried out in the Mediterranean alpine communities of Sierra de Guadarrama, a mountain range located in the Iberian Central System in Spain (40° 50′N, 3° 57′W), between 2000 and 2430 m.a.s.l. In these communities, pastures are dominated by graminoid *Festuca curvifolia* Lag. ex Lange and other perennial plants interspersed in a shrub matrix characterized by *Cytisus oromediterraneus* Rivas Mart*. et al*. and *Juniperus communis* subsp. *alpina* (Suter) Čelak. Butterflies (Order: Lepidoptera) are the predominant flower visitors followed by flies and hoverflies (Diptera) and large and small solitary bees (Hymenoptera)^6^.

### Sampling design and field survey

We replicated the study at two sites with similar characteristics representative of the Mediterranean alpine communities (Peñalara and Nevero peaks, Fig. S4 in Supporting Information). Site selection was based on the variables altitude, orientation, temperature and precipitation^6^. After snowmelt, we set up two 60 × 100 m sampling plots at each study site and made 20 line transects walks across the width of the sampling plots (60 m long and 5 m wide). We collected the identity and number of contacts between plant and insect species by making successive walks along the line transects. Transect methods are very effective for monitoring plant-flower visitor interactions in environments where time available for observations is limited^23^, as in the case of alpine environments. We recorded a plant-flower visitor interaction when an insect maintained contact with the reproductive organs of a flower for more than 1 second^23^. Thus, all flowering visiting insects that feed on flowers were recorded, regardless of the efficacy of their visit. Field sampling started when the first plant species of the community bloomed (*Armeria caespitosa* Boiss.) and continued until the end of the flowering period of the last species (*Silene ciliata* Pourr.)^6^. Weather conditions determined the number and distribution of census days, as warm, dry and light wind conditions are unusual in alpine environments^16^. Observations were made, from 10:00 to 18:00 h, when environmental conditions at these altitudes allowed pollination activity. They were carried out simultaneously in both sampling plots by a two-member team in each plot. We collected data from 14 June to 28 July for a total of 10 census days for each sampling site at Nevero (hereafter, NEV) and 11 census days for each sampling site at Peñalara (hereafter, PEN). There was one additional census day at PEN to obtain a similar number of survey hours at the two sites (approximately 160 hours per site). Flowering visiting insects were captured and determined to the lowest taxonomic category possible with the help of experts (Table S1). Voucher specimens were deposited at Rey Juan Carlos University. We grouped flower visitors into eleven functional groups following Lara‐Romero, *et al*.^6^ to facilitate the detection of general patterns (Appendix 1 and Table S1). Plant species were determined easily in the field. Once a week, the number of flowering individuals of each species was recorded in 10 transects (60 m long and 2 m wide) per study plot. The sum of all transects was used as an estimation of the total number of flowering plants per species at each site.

### Data analysis

#### Network construction and partition

We built cumulative quantitative bipartite networks for each study site. We used visitation frequencies, defined as the total number of visits of flower visitor *i* to plant species *j*, as a surrogate for interaction strength^39^. We also generated subnetworks as time-aggregated networks^7^ to analyse temporal dynamics in the flowering season. This methodology is useful to study structural changes based on the way participant species and their interactions change through time^7,8,26^. We broke down the cumulative networks into ten and eleven sub-networks for NEV and PEN, respectively, with each subnetwork spanning one census day.

#### Within-season variation in the composition of species assemblages and interactions

Within-season variation in the activity (*i.e*., number of visits) of each plant species and each functional group was visualized using spindle diagrams^40^. We applied beta diversity (variation of the species composition of assemblages) analysis to evaluate the dissimilarities among subnetworks, with each subnetwork spanning one census day, in a similar way to how classical studies of beta diversity use species communities at different sites. This methodology is useful to study structural changes based on the way participant species and their interactions change through time^7,8,26^. Thus, we applied the multiple-site beta diversity measures proposed by Ensing and Pither^41^ (see Table 2 therein for equations) to calculate the overall beta diversity of plant and flower-visitor species using Jaccard dissimilarity (β_CC_) and the proportion of replacement (β_3M_) and richness (β_RICH_) component. We used *R* code provided by Ensing and Pither^41^ for calculating multiple-site measures. We preferred this measure with respect to others because it can be transposed into a multisite approach^41^, thus allowing for an integrated view of the temporal dynamics of species. The replacement component of beta diversity is related to replacement of some species by others when we move from one-time period to another because of certain factors (e.g., variation in species phenology in our temporal perspective). On the other hand, the richness component of beta diversity is related to non-random species activity losses in a certain period, resulting in less rich biotas that are subsets of biotas in other time periods (i.e., at the time where most species are active in our temporal perspective). We expected temporal replacement of species would be constrained by the short flowering period (Fig. 1A) and, therefore, that overall β_CC_ would be low with a higher proportion of β_RICH_ at the expense of β_3M_.

Jaccard dissimilarity was also used to estimate dissimilarity in flower-visitors’ assemblages for all pairs of plant species comparisons. This measure was linearly regressed against inter-specific flowering overlap for assessing the existence of a relationship between flowering phenology and the similarity of interaction partners. The degree of overlap in flowering among species was calculated as S_*ij*_ = a_*ij*_/b_*ij*_, where a_ij_ is the number of days during which species *i* and *j* are in flower simultaneously, and b_*ij*_ is the number of days which at least one of them is in flower^42^.

Following Poisot, *et al*.^43^, dissimilarity in species (β_S_) and interactions (β_WN_) between pairwise combinations of networks was computed using Whittaker as the measure of dissimilarity:

$$\beta =\frac{a+b+c}{(2a+b+c)/2}-\,1;$$

where *a* is the number of species or interactions shared between two networks, *b* is the number present only in the first network, and *c* is the number present only in the second. This measure was selected because it incorporates differences in interaction composition related to richness changes and it has a value of 1 when sets are perfectly non-overlapping, and a value of 0 in case of perfect overlap, which can be directly translated into a pairwise distance between networks^43^.

We then calculated β_WN_ and β_S_ for every pairwise combination of time-aggregated networks using ‘betalink’ package in *R*^44^ and compared these metrics with the temporal distance between census days, using linear models. Then, if temporal replacement of species is constrained by the short flowering period of the study site (Fig. 1A), we would not expect a linear relationship between the distance among census days and dissimilarity of species and interactions.

#### Modularity analysis

Modularity was estimated for each cumulative network using the *QuaBiMo* algorithm (*Q*) implemented in *R* package *bipartite*^45^ which is based on a hierarchical random graph approach adapted for quantitative bipartite networks^12^. As the algorithm is a stochastic process results may vary among computations. For each network, we therefore ran the algorithm 100 times and retained the optimal modular configuration, *i.e*. the iteration with highest *Q* value^12^. We assessed the significance level of *Q* against a reference distribution derived from 100 random networks with the same species degree distribution as the empirical network^12^. Within-season variation in the activity of the identified modules (measured as total visits received by plants of each module) was visualized using spindle diagrams^40^. Additionally, we tested whether the activity of modules (measured as total visits received by plants of each module) varied in relation to time applying one-way χ^2^ tests^46^. In all tests, we computed *p*-value by a Monte Carlo simulation based on 5000 replications and applied the sequential Bonferroni correction to correct for multiple testing. To evaluate whether species within modules were organized according to their phenology, we used multinomial logistic regressions with module identity as response variable and start date of species activity as predictor variable. Multinomial logistic regressions allowed us to predict the probability of module membership based on species phenology. Likelihood ratio χ^2^ tests were used to evaluate goodness-of-fit of the models. Models were fitted using *R* package *nnet*^47^. For plants, start date of activity was defined as the number of days since 1 January until the first individual of the species initiates flowering, In the same way, start date of activity for flower visitors was the time until the first individual of the species was observed in a flower.

### Data Availability

The datasets generated during and/or analysed during the current study are available in the Dryad Digital Repository: 10.5061/dryad.p869n^19^.

## Electronic supplementary material

Supplementary information
