# Supplementary material for: Phenology drives species interactions and modularity in a plant - flower visitor network
Source: Sci Rep. 2018 Jun 20;8:9386. doi: 10.1038/s41598-018-27725-2 (PMC6010405; doi:10.1038/s41598-018-27725-2)
Supplement: Supplementary file 1 — Supplementary information [file 41598_2018_27725_MOESM1_ESM.pdf]

SUPPORTING INFORMATION

Phenology drives species interactions and modularity in a plant - flower visitor network

Morente-López J, Lara-Romero C, Ornosa C, Iriondo JM

Appendix 1. Functional groups of pollinators defined following the criteria of similarity in size, proboscis length, foraging behavior and feeding habits.

(i) Bumblebees, pollen-and nectar-collecting members of the bee genus Bombus;

(ii) Bees, mostly pollen- and nectar-collecting females measuring 7 mm in body length or larger;

(iii) Small bees, mostly pollen- and/or nectar-collecting females smaller than 7 mm;

(iv) Wasps, including aculeate wasps, large parasitic wasps collecting only nectar;

(v) Bee flies, long-tongued nectar-collecting Bombyliidae;

(vi) Hoverflies, nectar- and pollen-collecting Syrphidae and short-tongued Bombyliidae;

(vii) Flies, including nectar- and pollen- feeding species mainly belonging to the families Tachinidae and Muscidae;

(viii) Small flies, including feeding nectar- and/or pollen-feeding species smaller than 5 mm;

(ix) Beetles, including species collecting nectar and/or pollen;

(x) Butterflies (including diurnal moths), all nectar collectors;

(xi) Others, including nectar-collecting ants, small parasitic wasps, Microlepidoptera (micromoths), bugs and other occasional flower visitors.

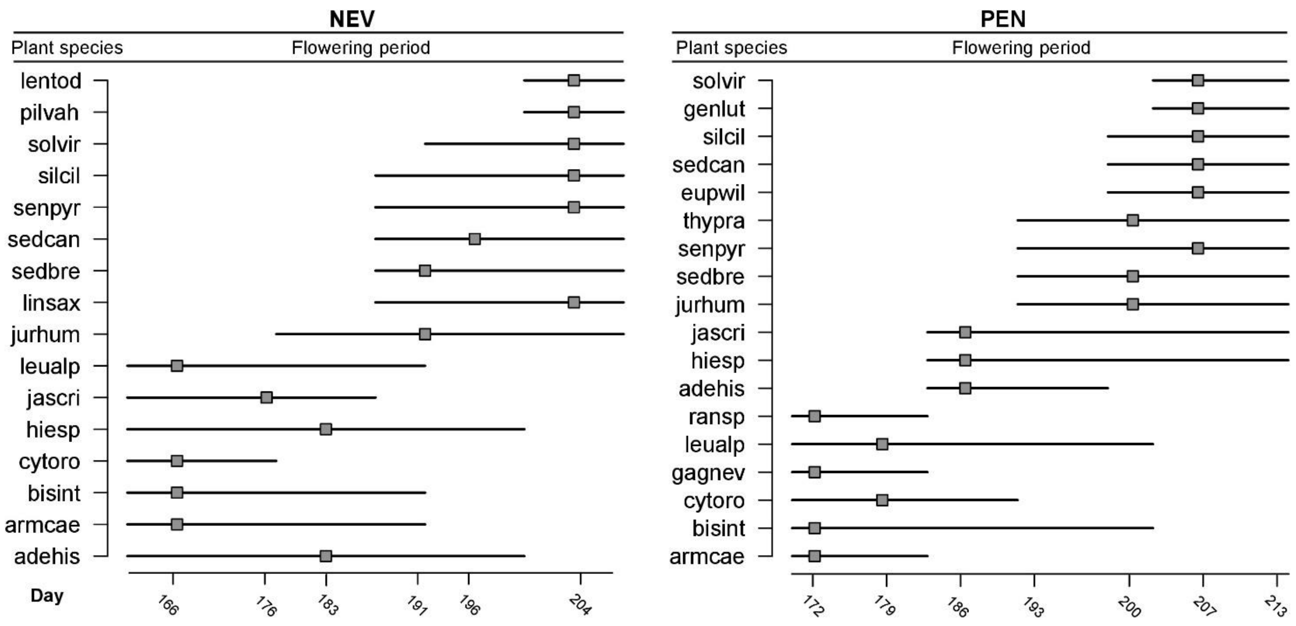

Fig. S1. Flowering phenology at Nevero and Peñalara study sites from 16 June to 28 July 2011. Lines indicate flowering period and squares represent the flowering peak. Ticks at X-axis denote time of sampling. Table S1 contains acronyms used for plant species. The end of the line after day 204 and 213 in NEV and PEN represents the end of the phenological study but not the end of the flowering period.

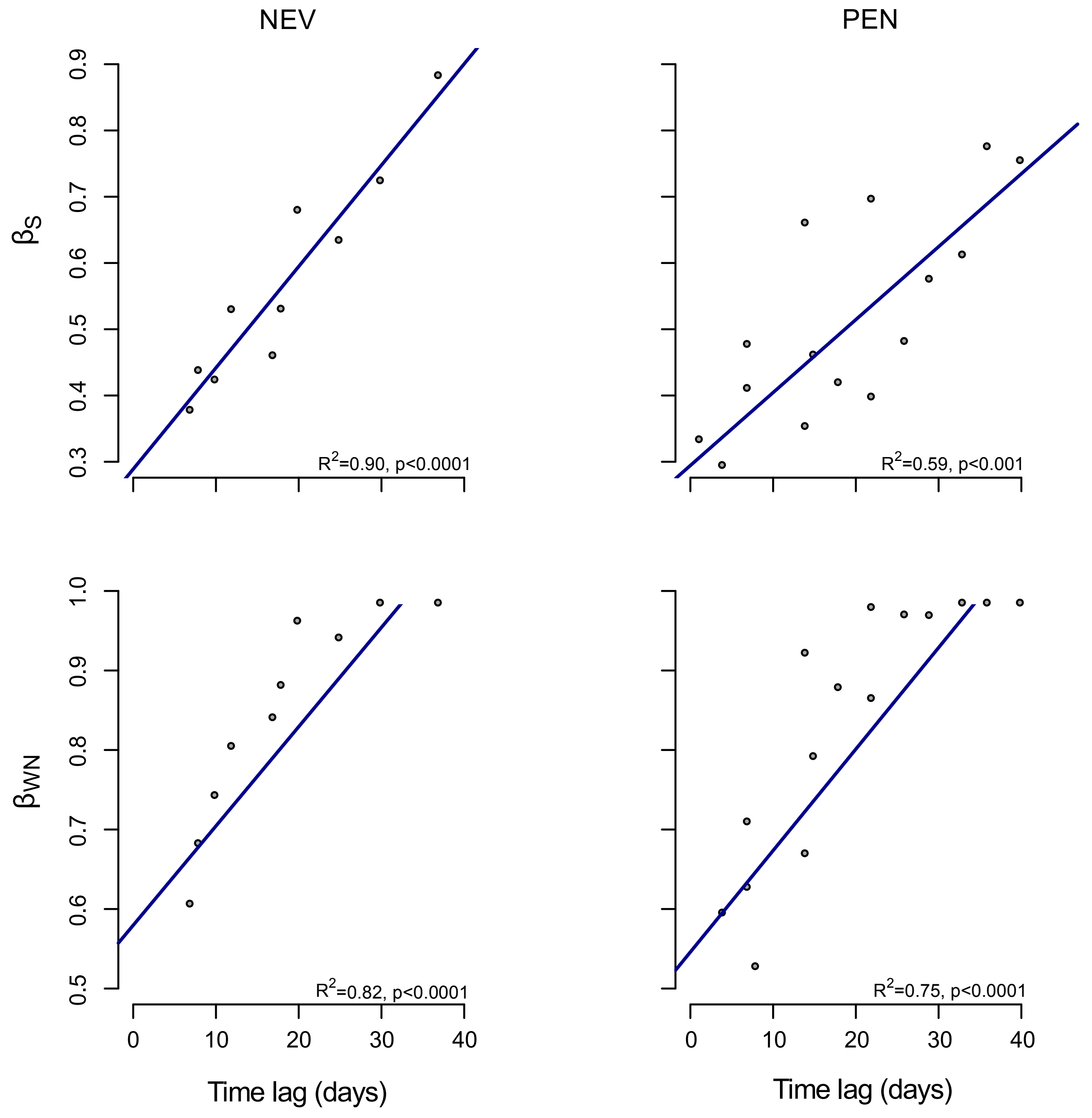

Figure S2. (A). Relationship between dissimilarity in species composition (β_S) and interactions (β_{WN}) and time lag between time-aggregated sub-networks with each aggregation time-window spanning two consecutive census days.

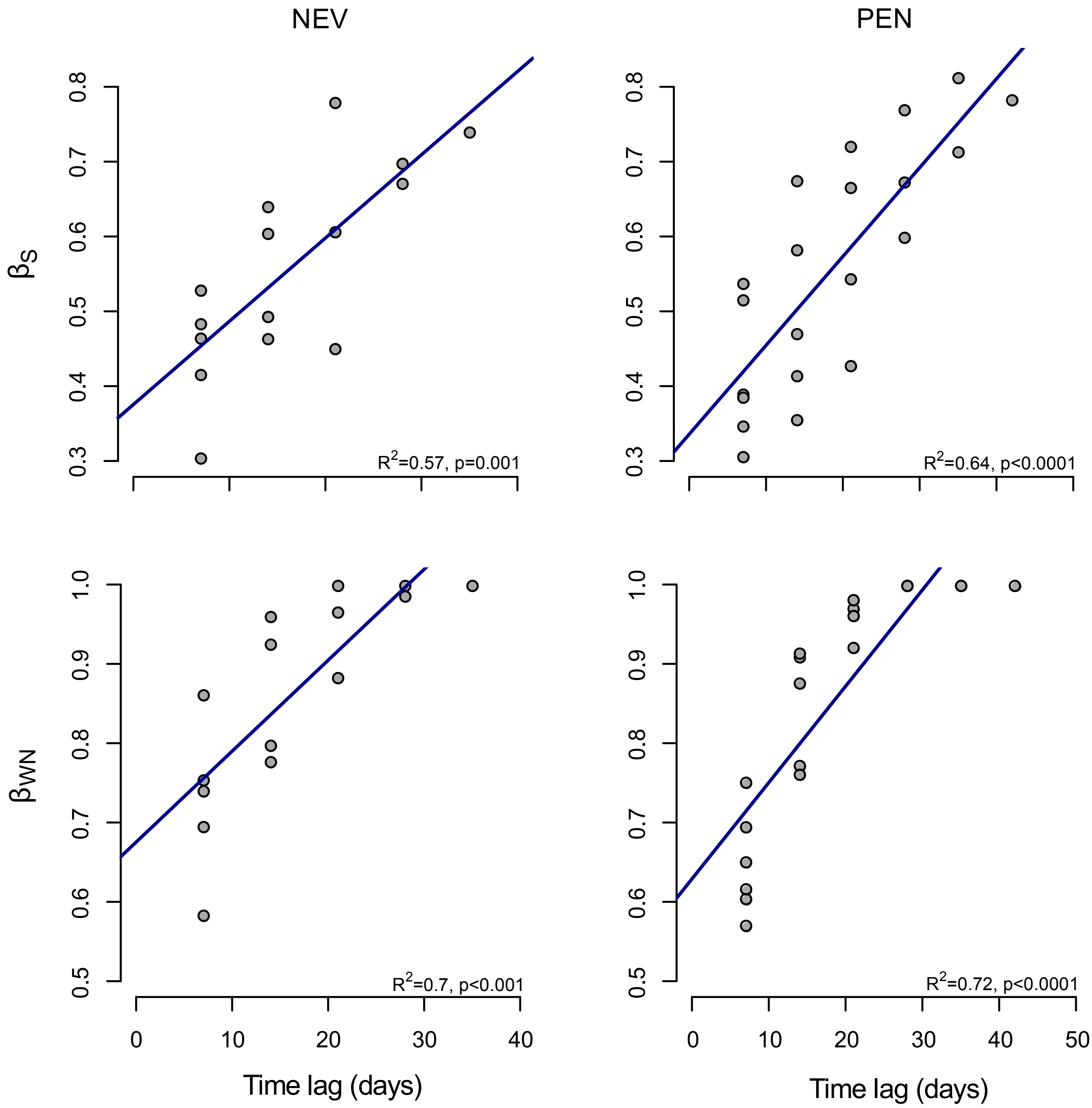

Figure S2. (B). Relationship between dissimilarity in species composition (β_S) and interactions (β_{WN}) and time lag between time-aggregated sub-networks with each aggregation time-window spanning a calendar week.

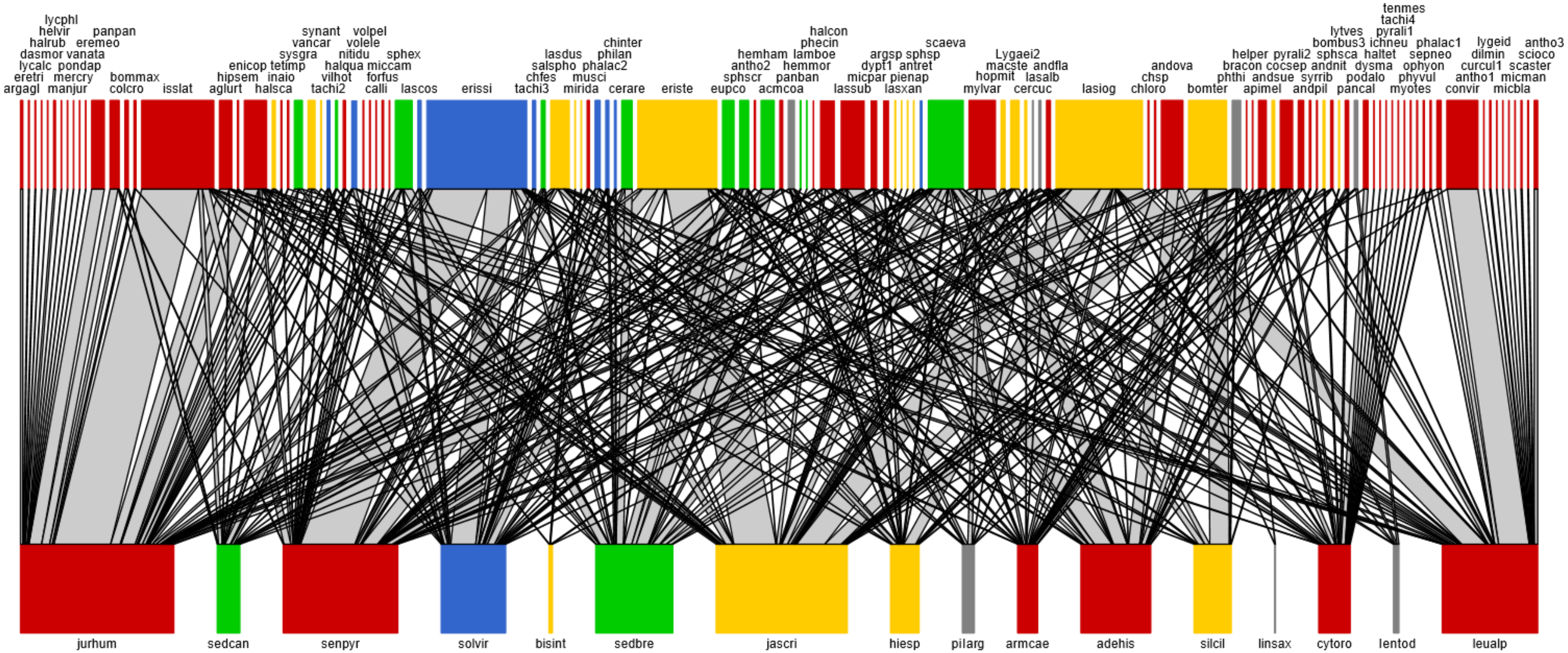

Figure S3 (A). Visitation networks from NEV. The colour of each rectangle represents the module to which the species belongs. Colour code is the same as in the Fig. 5. Red, NEV1; yellow, NEV2; green, NEV3; gray, NEV4, blue, NEV5. Table S1 contains acronyms used for species.

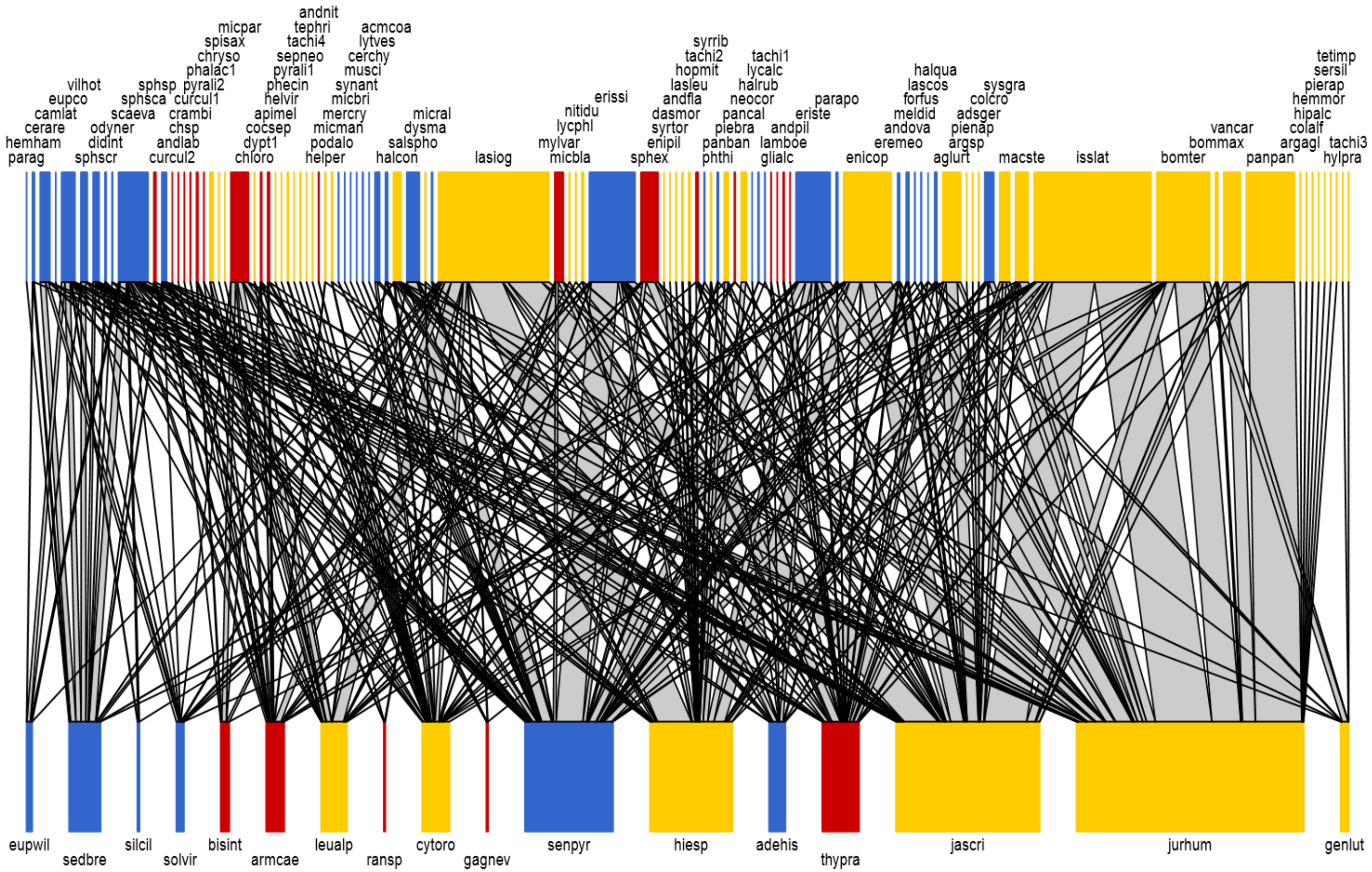

Figure S3 (B). Visitation networks from PEN. The colour of each rectangle represents the module to which the species belongs. Colour code is the same as in the Fig. 5. Red, NEV1; yellow, NEV2; blue, NEV3. Table S1 contains acronyms used for species.

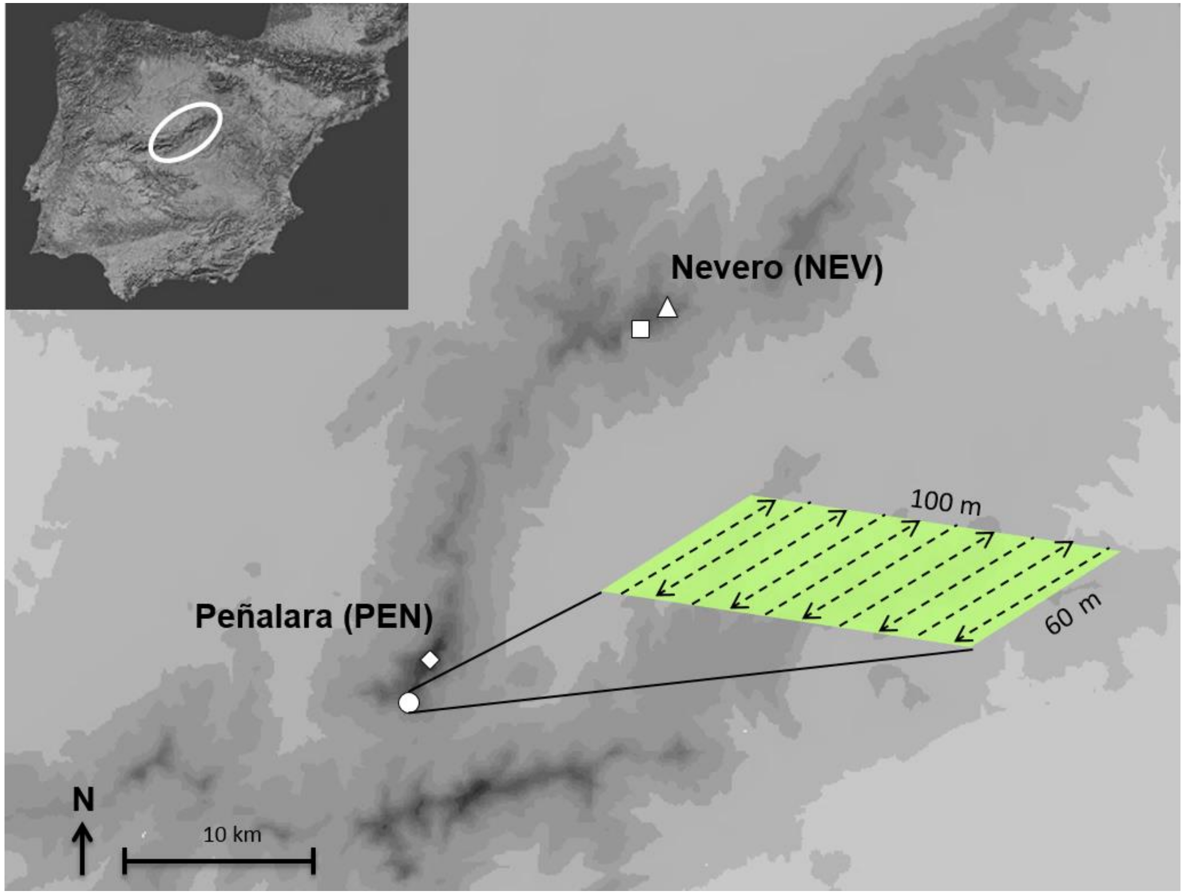

Fig. S4. Location of the sampling sites in Sierra de Guadarrama (Spain). Darker-shaded areas show higher elevation. Upper left map indicates the location of Sierra de Guadarrama in the Iberian Peninsula. Sampling plots were located at 2264 m (Peñalara-Diamond), 2214 m (Peñalara-Circle), 2202 m (Nevero-Triangle) and 2126 m (Nevero-Square) above sea level. Figure on the right side shows schematic drawing of the sampling procedure. 20 linear transects across the width of two 60 x 100m sampling plot were established at each sampling site. The map was created using Quantum GIS Desktop 2.14.1 (http://www.qgis.org/es/site).

Table S1. List of species studied in each study site and module. Acronym, scientific name, family, functional group and number of interactions and total number of visits recorded are indicated for each species.

Acronym	Species	Family	Group	Interactions	Visits	Module	Site
acmcoa	*Acmaeoderella coarctata coarctata* (Lucas, 1846)	Buprestidae	Beetles	4	7	NEV 1	NEV
adehis	*Adenocarpus hispanicus* (Lam.) DC.	Fabaceae	Plant	20	182	NEV 1	NEV
aglurt	*Aglais urticae* (Linneo, 1758)	Nymphalidae	Butterflies	6	32	NEV 1	NEV
andfla	*Andrena flavipes* Panzer, 1799	Andrenidae	Bees	6	10	NEV 1	NEV
andova	*Andrena ovatula* (Kirby 1802)	Andrenidae	Bees	4	56	NEV 1	NEV
andpil	*Andrena pilipes* Fabricius, 1781	Andrenidae	Bees	1	4	NEV 1	NEV
antho1	Anthomyiidae1	Anthomyiidae	Small flies	1	1	NEV 1	NEV
antho2	Anthomyiidae2	Anthomyiidae	Small flies	2	3	NEV 1	NEV
antho3	Anthomyiidae3	Anthomyiidae	Small flies	1	9	NEV 1	NEV
apimel	*Apis melifera* Linnaeus, 1758	Apidae	Bees	6	20	NEV 1	NEV
argagl	*Argynis aglaja* (Linnaeus, 1758)	Nymphalidae	Butterflies	1	6	NEV 1	NEV
armcae	*Armeria caespitosa* (Gómez Ortega) Boiss.	Plumbaginaceae	Plant	22	52	NEV 1	NEV
bommax	*Bombus maxillosus* Klug, 1817	Apidae	Bombus	2	5	NEV 1	NEV
bracon	Braconidae	Braconidae	Wasps	1	1	NEV 1	NEV
calli	Calliphoridae	Calliphoridae	Flies	1	1	NEV 1	NEV
chloro	Chloropidae	Chloropidae	Small flies	2	2	NEV 1	NEV
chsp	*Chrysotoxum* sp. Meigen, 1803	Syrphidae	Hoverflies	3	3	NEV 1	NEV
cocsep	*Coccinella septempunctata* Linnaeus, 1758	Coccinellidae	Beetles	7	32	NEV 1	NEV
colcro	*Colias crocceus* Fourcroy, 1785	Pieridae	Butterflies	2	9	NEV 1	NEV
convir	*Conophorus virescens* (Fabricius, 1787)	Bombyliidae	Bee Flies	2	81	NEV 1	NEV
curcul1	Curculionidae1	Curculionidae	Beetles	1	3	NEV 1	NEV
cytoro	*Cytisus oromediterraneus* Rivas Mart. & al.	Fabaceae	Plant	27	82	NEV 1	NEV
dasmor	*Dasypoda morotei* Quilis, 1928	Melittidae	Bees	1	1	NEV 1	NEV
dilmin	*Dilophus minor* Strobl, 1900	Bibionidae	Flies	1	1	NEV 1	NEV
dypt1	*Megaselia* sp. Róndani, 1856	Phoridae &Carnidae	Small flies	5	16	NEV 1	NEV

Acronym	Species	Family	Group	Interactions	Visits	Module	Site
dysma	*Dysmachus* sp. Loew, 1860	Asilidae	Flies	1	1	NEV 1	NEV
enicop	*Enicopus* sp. Stephens, 1830	Dasytidae	Beetles	9	58	NEV 1	NEV
eremeo	*Erebia meolans* (Prunner, 1798)	Nymphalidae	Butterflies	1	33	NEV 1	NEV
eretri	*Erebia triaria* (Prunner, 1799)	Nymphalidae	Butterflies	1	2	NEV 1	NEV
forfus	*Formica fusca* Linnaeus 1758	Formicidae	Others	1	4	NEV 1	NEV
halcon	*Halictus confusus* Smith, 1853	Halictidae	Bees	7	39	NEV 1	NEV
halqua	*Halictus quadricinctus* (Fabricius, 1776)	Halictidae	Bees	3	6	NEV 1	NEV
halrub	*Halictus rubicundus* (Christ, 1791)	Halictidae	Bees	1	1	NEV 1	NEV
haltet	*Halictus tetrazonius* (Klug, 1817)	Halictidae	Bees	1	1	NEV 1	NEV
helper	*Heliopathes perrouidi* (Mulsan & Rey, 1854)	Tenebrionidae	Beetles	1	1	NEV 1	NEV
helvir	*Heliothis viriplaca* (Hufnagel, 1766)	Noctuidae	Butterflies	1	5	NEV 1	NEV
hipsem	*Hipparchia semele* Linnaeus, 1758	Nymphalidae	Butterflies	2	3	NEV 1	NEV
hylpra	*Hylaeus praenotatus* (Förster, 1871)	Colletidae	Bees	1	1	NEV 1	NEV
inaio	*Inachis io* Linnaeus, 1759	Nymphalidae	Butterflies	2	2	NEV 1	NEV
isslat	*Issoria lathonia* (Linnaeus, 1758)	Nymphalidae	Butterflies	8	188	NEV 1	NEV
jurhum	*Jurinea humilis* (Desf.) DC.	Compositae	Plant	45	400	NEV 1	NEV
lassub	*Lasioglossum subfasciatum* (Imhoff, 1832)	Halictidae	Small Bees	5	63	NEV 1	NEV
leualp	*Leucanthemopsis alpina* (L.) Heywood	Compositae	Plant	35	249	NEV 1	NEV
lycalc	*Lycaena alciphron* (Rottemburg, 1775)	Lycaenidae	Butterflies	1	2	NEV 1	NEV
lycphl	*Lycaena phlaeas* (Linnaeus, 1761)	Lycaenidae	Butterflies	1	1	NEV 1	NEV
lygeid	Lygeidae	Lygeidae	Others	1	1	NEV 1	NEV
lytves	*Lytta vesicatoria* (Linnaeus, 1758)	Meloidae	Beetles	2	9	NEV 1	NEV
manjur	*Maniola jurtina* (Linnaeus, 1758)	Nymphalidae	Butterflies	1	1	NEV 1	NEV
mercry	*Merodon crypticus* Marcos-García, Vujic & Mengual, 2007	Syrphidae	Hoverflies	1	1	NEV 1	NEV
micbla	Microlepidoptera (micromoth) 1	-	Others	1	1	NEV 1	NEV
miccam	Microlepidoptera (micromoth) 3	-	Others	1	1	NEV 1	NEV

Acronym	Species	Family	Group	Interactions	Visits	Module	Site
micman	Microlepidoptera (micromoth) 4	-	Others	1	1	NEV 1	NEV
micpar	Microlepidoptera (micromoth) 5	-	Others	7	18	NEV 1	NEV
musci	Muscidae	Muscidae	Flies	4	6	NEV 1	NEV
mylvar	*Mylabris variabilis* (Pallas, 1781)	Meloidae	Beetles	4	69	NEV 1	NEV
myotes	*Myopa testacea* (Linneaus 1767)	Conopidae	Small flies	1	2	NEV 1	NEV
ophyon	*Ophyon* Fabricius, 1798	Ichneumonidae	Wasps	3	3	NEV 1	NEV
panpan	*Pandoriana pandora* (Denis & Schiffermüller, 1775)	Nymphalidae	Butterflies	3	24	NEV 1	NEV
phalac1	Phalacridae1	Phalacridae	Beetles	2	11	NEV 1	NEV
phecin	*Pherbellia cinerella* (Fallén, 1820)	Sciomyzidae	Small flies	3	4	NEV 1	NEV
phyvul	*Phratora vulgatissima* (Linnaeus, 1758)	Chrysomelidae	Beetles	1	2	NEV 1	NEV
podalo	*Podalonia tydei* Le Guillou, 1842 & *Podalonia* sp. (Fernald, 1927)	Sphecidae	Wasps	2	12	NEV 1	NEV
pondap	*Pontia daplidice* (Linnaeus, 1758)	Pieridae	Butterflies	1	1	NEV 1	NEV
pyrali1	Pyralidae1	Pyralidae	Butterflies	1	1	NEV 1	NEV
pyrali2	Pyralidae2	Pyralidae	Butterflies	3	14	NEV 1	NEV
scaster	*Scathophaga stercoraria* (Linnaeus, 1758)	Scathophagidae	Small flies	1	3	NEV 1	NEV
scioco	*Sciocoris sp.* Fallén, 1829	Pentatomidae	Others	1	2	NEV 1	NEV
senpyr	*Senecio pyrenaicus* L.	Compositae	Plant	39	299	NEV 1	NEV
sepneo	*Sepsis neocynipsea* Melander & Spuler, 1917	Sepsidae	Small flies	2	3	NEV 1	NEV
sphsca	*Sphecodes scabricollis* Wesmael, 1865	Halictidae	Small Bees	4	6	NEV 1	NEV
syrrib	*Syrphus ribesii* (Linnaeus, 1758)	Syrphidae	Hoverflies	1	2	NEV 1	NEV
tachi4	Tachinidae4	Tachinidae	Flies	1	1	NEV 1	NEV
tenmes	*Tenthredo mesomela* Linnaeus, 1758	Sphecidae	Wasps	1	1	NEV 1	NEV
tetimp	*Tetramorium impurum* (Förster, 1850)	Formicidae	Others	2	3	NEV 1	NEV
vanata	*Vanessa atalanta* (Linnaeus, 1758)	Nymphalidae	Butterflies	1	1	NEV 1	NEV
volele	*Volucella elegans* Loew, 1862	Syrphidae	Hoverflies	1	2	NEV 1	NEV

Acronym	*Species*	Family	Group	Interactions	Visits	Module	Site
volpel	*Volucella pellucens* (Linnaeus, 1758)	Syrphidae	Hoverflies	1	2	NEV 1	NEV
andnit	*Andrena nitida* (Müller, 1776)	Andrenidae	Bees	2	5	NEV 2	NEV
andsue	*Andrena suerinensis* Friese 1884	Andrenidae	Bees	3	7	NEV 2	NEV
antret	*Anthophora retusa* (Linnaeus, 1758)	Apidae	Bees	1	1	NEV 2	NEV
argsp	*Argynis* sp. (Rafinesque 1815)	Nymphalidae	Butterflies	1	2	NEV 2	NEV
bisint	*Biscutella intermedia* (Boiss. & Reut.) Malag.	Cruciferae	Plant	5	9	NEV 2	NEV
bombus	*Bombus* sp. (Latreille, 1802)	Apidae	Bombus	1	2	NEV 2	NEV
bomter	*Bombus terrestris* (Linnaeus, 1758)	Apidae	Bombus	4	99	NEV 2	NEV
eriste	*Eristalis tenax* (Linnaeus, 1758)	Syrphidae	Hoverflies	9	206	NEV 2	NEV
halsca	*Halictus scabiosae* (Rossi 1790)	Halictidae	Bees	2	8	NEV 2	NEV
hiesp	*Pilosella vahlii* (Froel.) F.W. Sch. & Sch. Bip.	Compositae	Plant	18	74	NEV 2	NEV
hopmit	*Hoplitis mitis* (Nylander, 1852)	Megachilidae	Bees	2	10	NEV 2	NEV
jascri	*Jasione crispa* (Pourr.) Samp.	Campanulaceae	Plant	34	342	NEV 2	NEV
lasdus	*Lasioglossum dusmeti* (Blüghten, 1924)	Halictidae	Small Bees	1	1	NEV 2	NEV
lasiog	*Lasioglossum leucopus* (Kirby 1802) & *L. cupromicans* (Pérez, 1903)	Halictidae	Small Bees	14	225	NEV 2	NEV
lasxan	*Lasioglossum xanthopus* (Kirby, 1802)	Halictidae	Small Bees	1	1	NEV 2	NEV
lygaei2	Lygaeidae	Lygeidae	Others	2	5	NEV 2	NEV
macste	*Macroglossum stellatarum* (Linnaeus, 1758)	Sphingidae	Butterflies	5	22	NEV 2	NEV
mirida	Miridae	Miridae	Others	1	1	NEV 2	NEV
pienap	*Pieris napi* (Linnaeus, 1758)	Pieridae	Butterflies	1	1	NEV 2	NEV
salspho	*Saltella sphondylii* (Schrank, 1803)	Sepsidae	Small flies	9	48	NEV 2	NEV
silcil	*Silene ciliata* Pourr.	Caryophyllaceae	Plant	9	97	NEV 2	NEV
synant	*Synanthedon sp*. Hübner 1819	Sesiidae	Butterflies	2	2	NEV 2	NEV
vancar	*Vanessa cardui* (Linnaeus, 1758)	Nymphalidae	Butterflies	3	18	NEV 2	NEV

Acronym	*Species*	Family	Group	Interactions	Visits	Module	Site
cerare	*Cerceris arenaria* (Linnaeus, 1758)	Crabronidae	Wasps	3	27	NEV 3	NEV
chfes	*Chrysotoxum festivum* (Linnaeus, 1758)	Syrphidae	Hoverflies	3	10	NEV 3	NEV
eupco	*Eupeodes corollae* (Fabricius, 1794)	Syrphidae	Hoverflies	6	30	NEV 3	NEV
hemham	*Hemipenthes* sp. Loew, 1854	Bombyliidae	Bee Flies	3	34	NEV 3	NEV
hemmor	*Hemipenthes morio* (Linnaeus, 1758)	Bombyliidae	Bee Flies	1	2	NEV 3	NEV
lamboe	*Lampides boeticus* (Linnaeus, 1767)	Lycaenidae	Butterflies	1	1	NEV 3	NEV
scaeva	*Scaeva albomaculata* (Macquart, 1842) & *S.-pyrastri* (Linnaeus, 1758)	Syrphidae	Hoverflies	12	91	NEV 3	NEV
sedbre	*Sedum brevifolium* DC.	Crassulaceae	Plant	24	201	NEV 3	NEV
Sedcan	*Sedum candollei* Raym.	Crassulaceae	Plant	19	59	NEV 3	NEV
sphex	*Sphex* sp. Linnaeus, 1758	Sphecidae	Wasps	5	44	NEV 3	NEV
sphscr	*Sphaeroforia scripta* (Linnaeus, 1758)	Syrphidae	Hoverflies	6	23	NEV 3	NEV
sysgra	*Systoechus gradatus* (Wiedemann in Meigen, 1820)	Bombyliidae	Bee Flies	4	21	NEV 3	NEV
vilhot	*Villa hottentotta* (Linnaeus, 1758)	Bombyliidae	Bee Flies	3	5	NEV 3	NEV
cercuc	*Ceratina cucurbitina* Rossi, 1792	Apidae	Small Bees	2	2	NEV 4	NEV
lasalb	*Lasioglossum albipes* (Fabricius, 1781)	Halictidae	Small Bees	5	5	NEV 4	NEV
lentod	*Lentodon* sp. L.	Compositae	Plant	6	14	NEV 4	NEV
linsax	*Linaria saxatalis* (L.) Chaz.	Scrophulariaceae	Plant	2	2	NEV 4	NEV
pancal	*Panurgus calcaratus* (Panzer 1807)	Panurginae	Small Bees	3	9	NEV 4	NEV
phthi	*Phthiria* sp. Meigen, 1820	Bombyliidae	Bee Flies	10	22	NEV 4	NEV
pilarg	*Pilosella argylocoma* (Fr.) F.W. Sch. & Sch. Bip.	Compositae	Plant	10	31	NEV 4	NEV
chinter	*Chrysotoxum intermedium* (Meigen, 1822)	Syrphidae	Hoverflies	2	4	NEV 5	NEV
erissi	*Eristalis similis* (Fallen, 1817)	Syrphidae	Hoverflies	11	261	NEV 5	NEV
lascos	*Lasioglossum costulatum* (Kriechbaumer, 1873)	Halictidae	Small Bees	3	8	NEV 5	NEV
nitidu	Nitidulidae	Nitidulidae	Beetles	5	16	NEV 5	NEV

Acronym	*Species*	Family	Group	Interactions	Visits	Module	Site
panban	*Panurgus banksianus* (Panzer 1806)	Panurginae	Small Bees	5	16	NEV 5	NEV
phalac2	Phalacridae2	Phalacridae	Beetles	5	13	NEV 5	NEV
philan	*Philanthus* sp. Fabricius, 1790	Sphecidae	Wasps	2	8	NEV 5	NEV
solvir	*Solidago virgaurea* L.	Compositae	Plant	25	168	NEV 5	NEV
sphsp	*Sphaerophoria* sp. Lepeletier & Serville, 1828	Syrphidae	Hoverflies	5	8	NEV 5	NEV
tachi2	Tachinidae2	Tachinidae	Flies	3	7	NEV 5	NEV
tachi3	Tachinidae3	Tachinidae	Flies	2	8	NEV 5	NEV
andlab	*Andrena labialis* (Kirby, 1802)	Andrenidae	Bees	1	1	PEN1	PEN
andpil	*Andrena pilipes* Fabricius, 1781	Andrenidae	Bees	1	1	PEN1	PEN
armcae	*Armeria caespitosa* (Gómez Ortega) Boiss.	Plumbaginaceae	Plant	17	81	PEN1	PEN
bisint	*Biscutella intermedia* (Boiss. & Reut.) Malag.	Cruciferae	Plant	6	39	PEN1	PEN
chsp	*Chrysotoxum* sp. Meigen, 1803	Syrphidae	Hoverflies	1	1	PEN1	PEN
cocsep	*Coccinella septempunctata* Linnaeus, 1758	Coccinellidae	Beetles	4	9	PEN1	PEN
crambi	Crambidae	Crambidae	Butterflies	1	2	PEN1	PEN
curcul1	Curculionidae1	Curculionidae	Beetles	3	7	PEN1	PEN
curcul2	Curculionidae2	Curculionidae	Beetles	1	1	PEN1	PEN
dypt1	*Megaselia* sp. Róndani, 1856	Phoridae &Carnidae	Small flies	5	6	PEN1	PEN
eremeo	*Erebia meolans* (Prunner, 1798)	Nymphalidae	Butterflies	5	16	PEN1	PEN
gagnev	*Gagea nevaensis* (Boiss.) O. Bolòs	Liliaceae	Plant	5	8	PEN1	PEN
glialc	-	-	Butterflies	1	1	PEN1	PEN
halsca	*Halictus scabiosae* (Rossi 1790)	Halictidae	Bees	2	4	PEN1	PEN
hopmit	*Hoplitis mitis* (Nylander, 1852)	Megachilidae	Bees	5	11	PEN1	PEN
lamboe	*Lampides boeticus* (Linnaeus, 1767)	Lycaenidae	Butterflies	1	5	PEN1	PEN
lascos	*Lasioglossum costulatum* (Kriechbaumer, 1873)	Halictidae	Small Bees	3	7	PEN1	PEN
micbla	Microlepidoptera (micromoth) 1	-	Others	2	3	PEN1	PEN

Acronym	*Species*	Family	Group	Interactions	Visits	Module	Site
micpar	Microlepidoptera (micromoth) 5	-	Others	9	79	PEN1	PEN
piebra	*Pieris brassicae* (Linnaeus, 1758)	Pieridae	Butterflies	3	5	PEN1	PEN
pyrali2	Pyralidae2	Pyralidae	Butterflies	2	2	PEN1	PEN
ransp	*Ranunculus* sp. L.	Ranunculaceae	Plant	4	7	PEN1	PEN
sphex	*Sphex* sp. Linnaeus, 1758	Sphecidae	Wasps	6	77	PEN1	PEN
sphsca	*Sphecodes scabricollis* Wesmael, 1865	Halictidae	Small Bees	4	10	PEN1	PEN
sysgra	*Systoechus gradatus* (Wiedemann in Meigen, 1820)	Bombyliidae	Bee Flies	3	45	PEN1	PEN
tachi1	Tachinidae1	Tachinidae	Flies	2	2	PEN1	PEN
thypra	*Thymus praecox* Opiz.	Labiatae	Plant	29	165	PEN1	PEN
adsger	*Adscita geryon* (Hübner 1813)	Zygaenidae	Butterflies	1	2	PEN2	PEN
aglurt	*Aglais urticae* (Linneo, 1758)	Nymphalidae	Butterflies	5	81	PEN2	PEN
andfla	*Andrena flavipes* Panzer, 1799	Andrenidae	Bees	1	4	PEN2	PEN
andnit	*Andrena nitida* (Müller, 1776)	Andrenidae	Bees	1	3	PEN2	PEN
apimel	*Apis melifera* Linnaeus, 1758	Apidae	Bees	1	1	PEN2	PEN
argagl	*Argynis aglaja* (Linnaeus, 1758)	Nymphalidae	Butterflies	1	1	PEN2	PEN
argsp	*Argynis* sp. (Rafinesque 1815)	Nymphalidae	Butterflies	1	1	PEN2	PEN
bommax	*Bombus maxillosus* Klug, 1817	Apidae	Bombus	3	11	PEN2	PEN
bomter	*Bombus terrestris* (Linnaeus, 1758)	Apidae	Bombus	9	236	PEN2	PEN
chloro	Chloropidae	Chloropidae	Small flies	2	2	PEN2	PEN
chryso	Chrysomelidae	Chrysomelidae	Beetles	1	1	PEN2	PEN
colalf	*Colias alfacariensis* Ribbe, 1905	Pieridae	Butterflies	1	2	PEN2	PEN
colcro	*Colias crocceus* Fourcroy, 1785	Pieridae	Butterflies	4	41	PEN2	PEN
cytoro	*Cytisus oromediterraneus* Rivas Mart. & al.	Fabaceae	Plant	27	123	PEN2	PEN
dasmor	*Dasypoda morotei* Quilis, 1928	Melittidae	Bees	1	2	PEN2	PEN
dysma	*Dysmachus* sp. Loew, 1860	Asilidae	Flies	2	3	PEN2	PEN
enicop	*Enicopus* sp. Stephens, 1830	Dasytidae	Beetles	8	212	PEN2	PEN

Acronym	*Species*	Family	Group	Interactions	Visits	Module	Site
enipil	*Enicopus pilosus* (Scopoli, 1763)	Dasytidae	Beetles	1	1	PEN2	PEN
genlut	*Gentiana lutea* L.	Gentianaceae	Plant	9	37	PEN2	PEN
halcon	*Halictus confusus* Smith, 1853	Halictidae	Bees	9	34	PEN2	PEN
helvir	*Heliothis viriplaca* (Hufnagel, 1766)	Noctuidae	Butterflies	1	1	PEN2	PEN
hemmor	*Hemipenthes morio* (Linnaeus, 1758)	Bombyliidae	Bee Flies	1	1	PEN2	PEN
hiesp	*Pilosella vahlii* (Froel.) F.W. Sch. & Sch. Bip.	Compositae	Plant	26	368	PEN2	PEN
hipalc	*Hipparchia alcyone* (Denis & Schiffermüller, 1775)	Nymphalidae	Butterflies	1	2	PEN2	PEN
hylpra	*Hylaeus praenotatus* (Förster, 1871)	Colletidae	Bees	1	1	PEN2	PEN
isslat	*Issoria lathonia* (Linnaeus, 1758)	Nymphalidae	Butterflies	11	522	PEN2	PEN
jascri	*Jasione crispa* (Pourr.) Samp.	Campanulaceae	Plant	40	642	PEN2	PEN
jurhum	*Jurinea humilis* (Desf.) DC.	Compositae	Plant	42	1014	PEN2	PEN
lasiog	*Lasioglossum leucopus* (Kirby 1802) & *L. cupromicans* (Pérez, 1903)	Halictidae	Small Bees	15	493	PEN2	PEN
lasleu	*Lasioglossum leucozonium* (Schrank, 1781)	Halictidae	Small Bees	1	6	PEN2	PEN
leualp	*Leucanthemopsis alpina* (L.) Heywood	Compositae	Plant	20	116	PEN2	PEN
lycphl	*Lycaena phlaeas* (Linnaeus, 1761)	Lycaenidae	Butterflies	2	2	PEN2	PEN
macste	*Macroglossum stellatarum* (Linnaeus, 1758)	Sphingidae	Butterflies	5	57	PEN2	PEN
nitidu	Nitidulidae	Nitidulidae	Beetles	3	8	PEN2	PEN
panban	*Panurgus banksianus* (Panzer 1806)	Panurginae	Small Bees	4	19	PEN2	PEN
pancal	*Panurgus calcaratus* (Panzer 1807)	Panurginae	Small Bees	2	23	PEN2	PEN
panpan	*Pandoriana pandora* (Denis & Schiffermüller, 1775)	Nymphalidae	Butterflies	4	215	PEN2	PEN
phalac1	Phalacridae1	Phalacridae	Beetles	2	14	PEN2	PEN
phecin	*Pherbellia cinerella* (Fallén, 1820)	Sciomyzidae	Small flies	1	4	PEN2	PEN
pienap	*Pieris napi* (Linnaeus, 1758)	Pieridae	Butterflies	1	1	PEN2	PEN
pierap	*Pieris rapae* (Linnaeus, 1758)	Pieridae	Butterflies	1	1	PEN2	PEN

Acronym	*Species*	Family	Group	Interactions	Visits	Module	Site
podalo	*Podalonia tydei* Le Guillou, 1842 & *Podalonia* sp. (Fernald, 1927)	Sphecidae	Wasps	2	5	PEN2	PEN
pyrali1	Pyralidae1	Pyralidae	Butterflies	1	1	PEN2	PEN
sepneo	*Sepsis neocynipsea* Melander & Spuler, 1917	Sepsidae	Small flies	1	3	PEN2	PEN
sersil	*Sericomyia silentis* (Harris, 1776)	Syrphidae	Hoverflies	1	1	PEN2	PEN
spisax	*Spilostethus saxatilis* (Scopoli, 1763)	Lygeidae	Others	1	1	PEN2	PEN
syrrib	*Syrphus ribesii* (Linnaeus, 1758)	Syrphidae	Hoverflies	2	4	PEN2	PEN
syrtor	*Syrphus torvus* Osten-Sacken, 1875	Syrphidae	Hoverflies	1	1	PEN2	PEN
tachi3	Tachinidae3	Tachinidae	Flies	1	1	PEN2	PEN
tachi4	Tachinidae4	Tachinidae	Flies	1	1	PEN2	PEN
tephri	Tephritidae	Tachinidae	Flies	1	1	PEN2	PEN
tetimp	*Tetramorium impurum* (Förster, 1850)	Formicidae	Others	1	1	PEN2	PEN
vancar	*Vanessa cardui* (Linnaeus, 1758)	Nymphalidae	Butterflies	2	75	PEN2	PEN
acmcoa	*Acmaeoderella coarctata coarctata* (Lucas, 1846)	Buprestidae	Beetles	3	10	PEN3	PEN
adehis	*Adenocarpus hispanicus* (Lam.) DC.	Fabaceae	Plant	14	73	PEN3	PEN
andova	*Andrena ovatula* (Kirby 1802)	Andrenidae	Bees	2	12	PEN3	PEN
camlat	*Camptopus lateralis* (German, 1817)	Alydidae	Others	1	1	PEN3	PEN
cerare	*Cerceris arenaria* (Linnaeus, 1758)	Crabronidae	Wasps	5	43	PEN3	PEN
cerchy	*Cerceris rhybyensis* (Linnaeus, 1771)	Crabronidae	Wasps	1	1	PEN3	PEN
didint	*Didea intermedia* Loew, 1854	Syrphidae	Hoverflies	2	6	PEN3	PEN
erissi	*Eristalis similis* (Fallen, 1817)	Syrphidae	Hoverflies	9	204	PEN3	PEN
eriste	*Eristalis tenax* (Linnaeus, 1758)	Syrphidae	Hoverflies	8	153	PEN3	PEN
eupco	*Eupeodes corollae* (Fabricius, 1794)	Syrphidae	Hoverflies	11	60	PEN3	PEN
eupwil	*Euphrasia willkommii* Freyn.	Scrophulariaceae	Plant	5	26	PEN3	PEN
forfus	*Formica fusca* Linnaeus 1758	Formicidae	Others	3	3	PEN3	PEN
halqua	*Halictus quadricinctus* (Fabricius, 1776)	Halictidae	Bees	5	10	PEN3	PEN
halrub	*Halictus rubicundus* (Christ, 1791)	Halictidae	Bees	2	4	PEN3	PEN

Acronym	*Species*	Family	Group	Interactions	Visits	Module	Site
hemham	*Hemipenthes* sp. Loew, 1854	Bombyliidae	Bee Flies	3	9	PEN3	PEN
lycalc	*Lycaena alciphron* (Rottemburg, 1775)	Lycaenidae	Butterflies	2	2	PEN3	PEN
lytves	*Lytta vesicatoria* (Linnaeus, 1758)	Meloidae	Beetles	2	21	PEN3	PEN
meldid	*Melitaea didyma* (Esper, 1779)	Nymphalidae	Butterflies	2	2	PEN3	PEN
mercry	*Merodon crypticus* Marcos-García, Vujic & Mengual, 2007	Syrphidae	Hoverflies	1	1	PEN3	PEN
micbri	Microlepidoptera (micromoth) 2	-	Others	1	1	PEN3	PEN
micman	Microlepidoptera (micromoth) 4	-	Others	1	2	PEN3	PEN
micral	Microlepidoptera (micromoth) 6	-	Others	2	5	PEN3	PEN
musci	Muscidae	Muscidae	Flies	1	3	PEN3	PEN
mylvar	*Mylabris variabilis* (Pallas, 1781)	Meloidae	Beetles	6	39	PEN3	PEN
neocor	*Neomyia cornicina* (Fabricius, 1781)	Muscidae	Flies	1	1	PEN3	PEN
odyner	*Odynerus* sp. (Latreille, 1802)	Eumenidae	Wasps	3	3	PEN3	PEN
parag	*Paragus sp.* Latreille, 1804	Syrphidae	Hoverflies	1	1	PEN3	PEN
parapo	*Parnassius apolo* (Linnaeus, 1758)	Papilionidae	Butterflies	3	8	PEN3	PEN
phthi	*Phthiria* sp. Meigen, 1820	Bombyliidae	Bee Flies	3	6	PEN3	PEN
salspho	*Saltella sphondylii* (Schrank, 1803)	Sepsidae	Small flies	4	56	PEN3	PEN
scaeva	*Scaeva albomaculata* (Macquart, 1842) & *S.-pyrastri* (Linnaeus, 1758)	Syrphidae	Hoverflies	13	132	PEN3	PEN
sedbre	*Sedum brevifolium* DC.	Crassulaceae	Plant	15	141	PEN3	PEN
senpyr	*Senecio pyrenaicus* L.	Compositae	Plant	40	394	PEN3	PEN
silcil	*Silene ciliata* Pourr.	Caryophyllaceae	Plant	3	10	PEN3	PEN
solvir	*Solidago virgaurea* L.	Compositae	Plant	13	34	PEN3	PEN
sphscr	*Sphaeroforia scripta* (Linnaeus, 1758)	Syrphidae	Hoverflies	9	27	PEN3	PEN
sphsp	*Sphaerophoria* sp. Lepeletier & Serville, 1828	Syrphidae	Hoverflies	9	21	PEN3	PEN
synant	*Synanthedon sp*. Hübner 1819	Sesiidae	Butterflies	1	1	PEN3	PEN
tachi2	Tachinidae2	Tachinidae	Flies	2	3	PEN3	PEN
vilhot	*Villa hottentotta* (Linnaeus, 1758)	Bombyliidae	Bee Flies	5	29	PEN3	PEN

Table S2. Logistic coefficients and standard errors (SE) fitted for each predictor variable and each module in Nevero and Peñalara study sites. Modules 1 in Nevero (NEV 1) and Peñalara (PEN 1) were used as reference

	Dependent variable (Module identity)			
Nevero (NEV)				
	NEV 2 (SE)	NEV 3 (SE)	NEV 4 (SE)	NEV 5 (SE)
Onset of activity	-0.329	0.081	0.049	0.079
	(0.025)	(0.026)	(0.03)	0.029)
Constant	4.525	-16.552	-11.037	-16.466
	(4.272)	(4.876)	(5.5199	(5.383)
Peñalara (PEN)				
	PEN 2 (SE)	PEN 3 (SE)		
Onset of activity	0.046	0.115		
	(0.026)	(0.028)		
Constant	-7.744	-21.036		
	(4.664)	(5.159)		

Table S3. Within-season beta-diversity of plant and flower visitor assemblages at Nevero (NEV) and Peñalara (PEN) study sites. Beta-diversity was analysed among sub-networks spanning two consecutive census days and a calendar week.

	Sub-networks spanning a calendar week (n=6 for NEV, n=7 for PEN)			Sub-networks spanning two consecutive census days (n=5 for NEV, n=6 for PEN)		
	β_{CC}	β_{3M}	β_{RICH}	β_{CC}	β_{3M}	β_{RICH}
Plant species assemblages						
NEV	0.78	0.49	0.29	0.74	0.52	0.22
PEN	0.82	0.69	0.13	0.76	0.62	0.14
Flower visitor assemblages						
NEV	0.83	0.60	0.23	0.79	0.69	0.10
PEN	0.85	0.60	0.25	0.82	0.64	0.18

B_{CC} is overall Jaccard dissimilarity. β_{3M} and β_{RICH} are replacement and richness component of Jaccard dissimilarity, respectively.
